# What can individual differences reveal about face processing?

**DOI:** 10.3389/fnhum.2014.00562

**Published:** 2014-08-19

**Authors:** Galit Yovel, Jeremy B. Wilmer, Brad Duchaine

**Affiliations:** ^1^School of Psychological Sciences, Sagol School of Neuroscience, Tel Aviv UniversityTel Aviv, Israel; ^2^Department of Psychology, Wellesley CollegeWellesley, MA, USA; ^3^Department of Psychological and Brain Sciences, Dartmouth CollegeHanover, NH, USA

**Keywords:** face recognition, individual differences, holistic processing, fusiform face area, behavioral genetics

## Abstract

Faces are probably the most widely studied visual stimulus. Most research on face processing has used a group-mean approach that averages behavioral or neural responses to faces across individuals and treats variance between individuals as noise. However, individual differences in face processing can provide valuable information that complements and extends findings from group-mean studies. Here we demonstrate that studies employing an individual differences approach—examining associations and dissociations across individuals—can answer fundamental questions about the way face processing operates. In particular these studies allow us to associate and dissociate the mechanisms involved in face processing, tie behavioral face processing mechanisms to neural mechanisms, link face processing to broader capacities and quantify developmental influences on face processing. The individual differences approach we illustrate here is a powerful method that should be further explored within the domain of face processing as well as fruitfully applied across the cognitive sciences.

The cognitive and neural bases of face processing have been extensively investigated. The majority of these studies have taken a group-mean approach, focusing on the average cognitive or neural response and treating natural variation across individuals as noise. Here, we seek to highlight a small but growing literature that treats such variation as a valuable signal in its own right. These studies complement and extend group-mean studies by providing a powerful, independent way to examine the functional organization, neural bases, and developmental origins of skilled face processing (Wilmer, 2008). As such, the goal of this review is not merely to document the existence of individual differences in face processing. Rather, we focus on cases where associations and dissociations across individuals have advanced our theoretical understanding. These associations and dissociations have, for example, tested theorized links between behavioral face processing measures such as measures of holistic processing or the processing of familiar and unfamiliar faces; they have associated and dissociated different neural face processing measures and examined their relationships with behavior; they have dissociated face recognition from more general cognitive abilities; and they have isolated genetic and environmental contributions to face processing.

## Are face recognition abilities mediated by holistic processing mechanisms?

**Figure 1:**
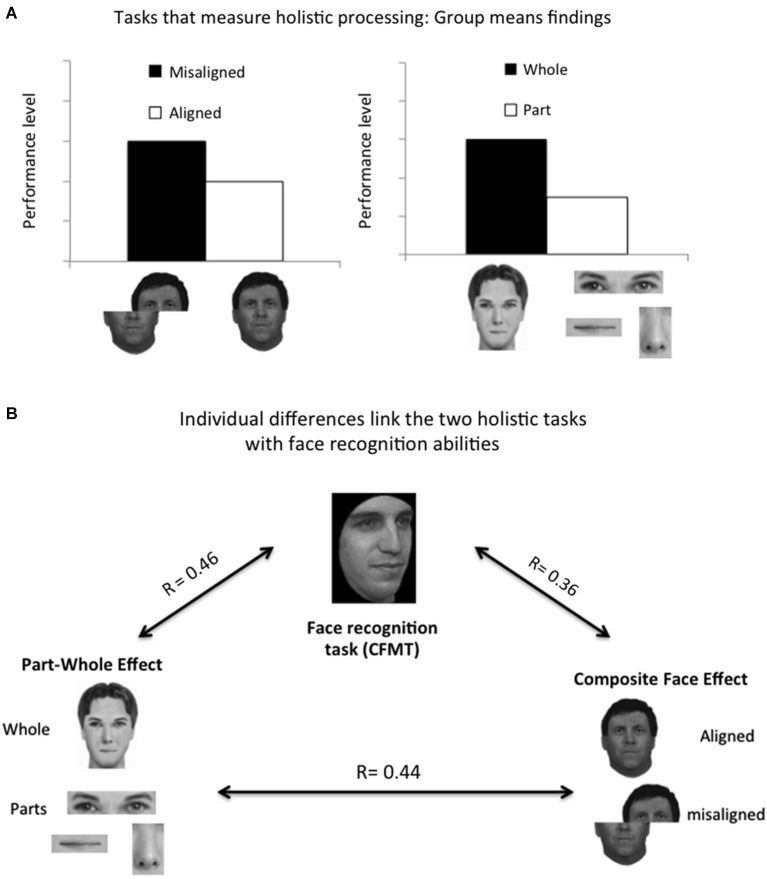
**(A)** Group mean findings of the classical holistic processing measures. The part-whole task shows better recognition of face parts when presented within the whole face (i.e., whole condition) than when presented in isolation (i.e., part condition). The composite face task shows better recognition of upper or lower part of the face when it is misaligned than when it is aligned with an another face of a different identity, indicating interactions (which leads to interference) between the two face halves in the aligned condition (see also footnote 1). **(B)** Correlational analyses between the two measures of holistic processing and their relationship with face recognition abilities reveal moderate correlations (Degutis et al., 2013; see also footnote 2).

The notion that faces are processed more holistically than objects is one of the most extensively studied ideas in the face processing literature. Whereas holistic processing has been defined in many different ways in the literature (Richler et al., 2012), it has been typically measured with two main tasks: the part-whole task (Tanaka and Farah, 1993) and the composite face task (Young et al., 1987; Le Grand et al., 2004; see Figure 1A). In the part-whole task, subjects are asked to recognize face parts of faces they were previously learned either when they are embedded in a whole face or when presented alone. Performance level is typically better when the parts are presented within a whole face than when presented alone (Figure 1A, right). The composite task compares recognition of one half of a face when the other irrelevant half is inconsistent. Recognition of one face half is better when the other inconsistent half is misaligned than when it is aligned (Figure 1A, left).

Both of these tasks have shown interactive processing among face parts for upright faces but little or no interactivity for inverted faces or non-face objects (for review see, Maurer et al., 2002). These findings have led to suggestions that it is this holistic processing ability that underlies our remarkable face recognition abilities (Maurer et al., 2002). Whereas several studies with prosopagnosic individuals indicated impaired holistic processing of faces as measured with the composite task (Le Grand et al., 2006; Avidan et al., 2011; Palermo et al., 2011) suggesting that poor face recognition abilities may be associated with impaired holistic processing, this question has not been examined in normal individuals until recent individual differences studies examined the correlation between holistic processing measures and face recognition abilities.

The first study that directly assessed the correlations between these tasks (Konar et al., 2010) measured the composite effect by subtracting performance on aligned (i.e., whole condition) and misaligned (i.e., part condition) faces. Surprisingly this measure of holistic processing failed to correlate with their measure of face recognition abilities. Three subsequent studies, however, have found significant positive relationships between face recognition and both the composite effect (Richler et al., 2011; Wang et al., 2012; Degutis et al., 2013) and the part-whole effect (Wang et al., 2012; Degutis et al., 2013) but not with performance on a non-face global local task (Wang et al., 2012) suggesting that these correlations are not mediated by general visual processing abilities. Two of these studies (Richler et al., 2011; Degutis et al., 2013) used the reliable and well-validated Cambridge Face Memory Test (CFMT; Wilmer et al., 2012), which may partially account for the higher correlations they revealed. These studies further found that the size of the correlations depends on the analytic method used to measure holistic processing (regression vs. subtraction scores, Degutis et al., 2013) and the design of the composite task used (congruency/interference vs. standard design, Richler and Gautheir, 2013; Rossion, 2013).^1^

Taken as a whole, these studies suggest that holistic processing and face recognition are indeed linked, but not as strongly as had been widely assumed; therefore, other factors must also contribute to face recognition abilities. One such factor was suggested by a recent study showing a small but significant correlation across individuals between face recognition ability and face aftereffects (Dennett et al., 2012). The face aftereffect is used as a tool to assess face space coding of face identity. The magnitude of the face aftereffect reflects the steepness of the response function along a given dimension (i.e., facial feature). Dennett et al. (2012) have revealed that steeper functions are associated with better face recognition abilities. Future studies are needed to assess the relative contributions of holistic processing, face space coding, and other factors to face recognition abilities.

## Do different measures of holistic face processing tap the same mechanisms?

Given evidence that faces are processed by holistic mechanisms, another basic question that has been overlooked for many years is whether different measures of holistic processing reflect the same holistic processing mechanism. The part-whole and the composite face effect have often been considered measures of the same process and have been used interchangeably in the face processing literature (Richler et al., 2012). It was therefore puzzling when studies revealed low correlations between these two holistic processing measures (*r* = 0.23 in Degutis et al., 2013; *r* = 0.03 in Wang et al., 2012). Degutis et al. (2013) however found that the correlation between the two holistic measures was substantial (*r* = 0.44) when holistic processing scores were computed via a regression-based method (Figure 1B) and were higher than when they were computed via the subtraction-based method used in group-mean studies; this finding has generated discussion of how to translate measures used in group-mean studies into a form that can validly capture both clinical and non-clinical human variation (for an extensive discussion of this question see Degutis et al., 2013)^2^. More generally, such individual differences based studies force us to critically examine commonly used measures and better determine what they measure.

## Are face parts and their spacing represented by the same mechanism or different mechanisms?

A study that examined individual differences in discrimination of face parts and with spacing between parts provides another example of how individual differences findings may not only complement data from group-means studies, but also aid in defining our measures of interest (Yovel and Kanwisher, 2008). The term configural processing has been frequently used to describe how faces are represented (Maurer et al., 2002). One way in which configural processing has been measured is by examining sensitivity to the distance among face parts (e.g., distance among the two eyes), which has been claimed to be critical for face recognition (Le Grand et al., 2001). However, more recent papers highlighted the role of both the spacing and the shape of their parts in face processing and suggested that they are both mediated by the same face processing mechanism (Yovel and Duchaine, 2006; McKone and Yovel, 2009; Amishav and Kimchi, 2010). An individual differences study strongly supported the latter claim, by showing a high correlation between discrimination of faces that differ in spacing among parts and faces that differ in the shape of parts for upright faces. In contrast, the same correlation was effectively zero for inverted faces or houses (Yovel and Kanwisher, 2008). These findings have led to the suggestion that the definition of holistic/configural processing should include the processing of both the shapes of parts and the spacing among them (McKone and Yovel, 2009). Consistent with these findings, Yovel and Duchaine (2006) have reported that prosopagnosic individuals show similarly poor discrimination of face parts and the spacing among them, which suggests both types of information are impaired in individuals with poor face recognition abilities.

## Do face expression and face identity processing rely on different mechanisms?

The question of whether face expression and identity are processed by a common mechanism or separate mechanisms has been debated in the cognitive (Ganel and Goshen-Gottstein, 2004), neuropsychological (Bruce and Young, 1986) and neuroimaging literature (Calder and Young, 2005; Gobbini and Haxby, 2007). An individual differences approach can address this question by assessing the correlations among tests of expression and identity processing. A critical prerequisite for such an approach, however, is reliable and valid tests of expression processing. In a recent study Palermo et al. (2011) argued that no existing expression processing test met the high standards necessary to enable such an approach. They then developed two new tests, one expression matching test and one expression labeling test. Both tests efficiently captured expression processing abilities, demonstrating strong reliability and validity despite their brevity; moreover, these tests demonstrated suitability for capturing a broad range of performance, avoiding the ceiling effects found in the majority of existing expression processing tests (Palermo et al., 2011). Interestingly, in an 80-person sample, the variation shared between these two expression recognition tests (*r* = 0.45) was virtually independent of performance on the CFMT (Duchaine and Nakayama, 2006), evidence for expression-specific mechanisms. At the same time, the expression matching test correlated robustly with the CFMT (*r* = 0.40), evidence for face-general mechanisms. Further studies are now needed to determine which particular expression processing mechanisms are shared with, and which are independent of, identity processing.

## Are familiar and unfamiliar faces processed by different mechanisms?

Face recognition abilities have been measured both with famous or personally familiar faces and with unfamiliar faces. In several studies Burton et al. provided evidence that familiar faces may be processed qualitatively differently from unfamiliar faces (Jenkins and Burton, 2011). They then used an individual differences approach to provide an independent test of their theory, examining the correlations between performance on several tasks that examined matching of upright and inverted familiar and unfamiliar faces (Megreya and Burton, 2006). Whereas correlations between tasks that measured unfamiliar face matching abilities were high, relatively low correlations were found between tasks that examined matching of unfamiliar and familiar faces. Interestingly, the correlation between matching of unfamiliar upright faces was highly correlated with matching of inverted familiar faces. Based on the notion that face processing mechanisms are specialized for the processing of upright but not inverted faces the authors interpret these findings as strong support for their theory of qualitatively different processing of familiar vs. unfamiliar faces, going so far as to suggest that “unfamiliar faces are not faces”. These findings illustrate how individual differences can provide an independent test of a theory derived from group-mean studies. It is noteworthy that unlike the lack of correlation found in matching tasks, correlations between famous and unfamiliar faces are found in a memory task (Wilmer et al., 2012). Furthermore, most prosopagnosic individuals are impaired on both familiar and unfamiliar face recognition tasks (Duchaine et al., 2007; Dalrymple et al., 2011). Future studies are now needed to determine whether these findings, both group-mean based and individual differences based, hold across a variety of face matching and face memory tasks.

## Do cognitive and neural measures of face processing reflect the same mechanisms?

**Figure 2:**
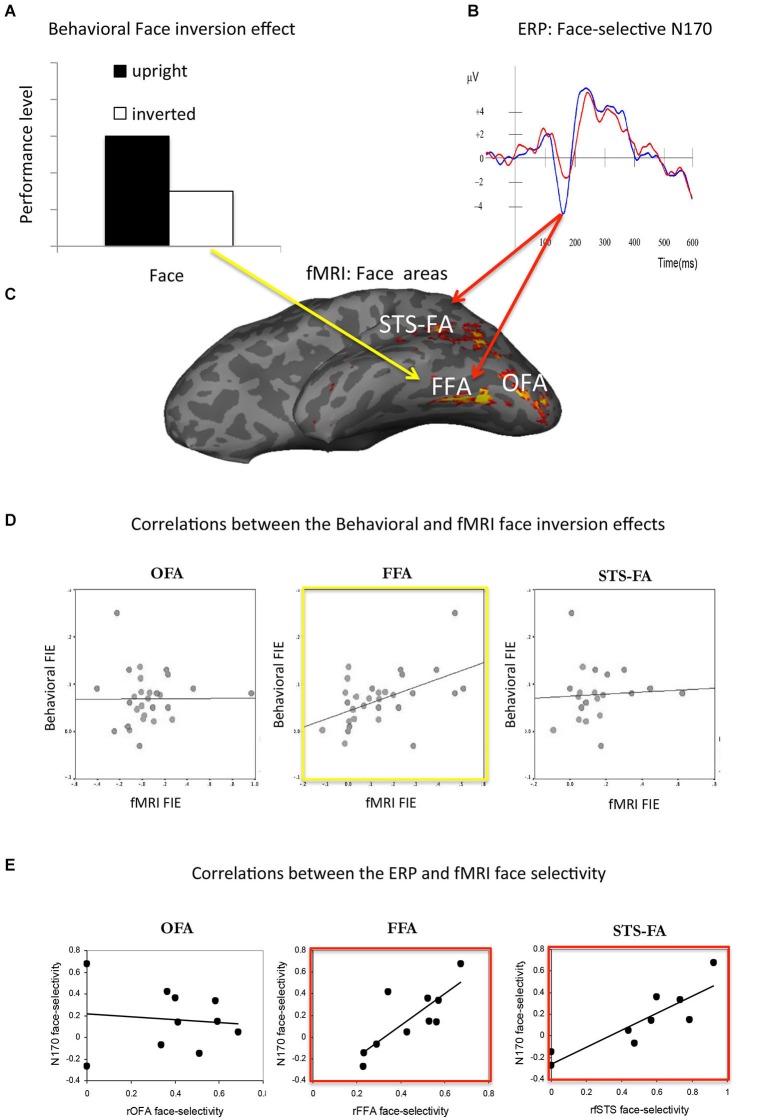
**(A)** The face inversion effect refers to the better recognition of faces presented in upright orientation than inverted (i.e., upside down). **(B)** The face N170: event-related potentials (ERPs) show higher amplitude to faces than non-face stimuli (i.e., face-selectivity) at 170 ms after stimulus onset. **(C)** Functional MRI studies reveal three face-selective (i.e., higher response to faces than non-face objects) regions in the occipital-temporal cortex: the occipital face area (OFA) in the lateral occipital cortex, the fusiform face area (FFA) in the fusiform gyrus and the superior temporal sulcus face area (STS-FA) in the posterior part of the STS. **(D)** The association between the behavioral face inversion effect (i.e., difference in performance level for upright than inverted faces) and the fMRI face inversion effect (i.e., difference in fMRI response to upright than inverted faces) was found only with the FFA (Yovel and Kanwisher, 2005). **(E)** Correlations among the magnitude of face selectivity (i.e., difference in ERP or fMRI response to faces than non-faces) was found at 170 ms with the FFA and STS-FA, but not with the OFA (Sadeh et al., 2010).

Faces are known to elicit robust and distinct neural responses with both functional MRI and electrophysiological measures (Figure 2). To better understand what type of processing these neural measures reflect, it is important to determine to what extent they are associated with cognitive measures of face processing as well as the extent to which different neural measures are correlated among themselves.

One of the most well-established findings in the face processing literature is the face inversion effect—that is the substantial drop in performance found for inverted relative to upright faces (Figure 2A, Yin, 1969). A difference between the group mean response to upright and inverted faces was found in two face areas, the fusiform face area (FFA) and the superior temporal sulcus face area (STS-FA) response was higher for upright than inverted faces, whereas in the lateral occipital complex (LOC) object area the response was higher for inverted than upright faces (Yovel and Kanwisher, 2005). However, a correlation between the behavioral and fMRI measures of the face inversion effect was found only with the FFA (Figure 2D). These findings suggest the FFA as a neural locus of the face inversion effect and highlight the importance of assessing correlations as well as differences in mean responses, because group means may be consistent with the behavioral effect but not associated with it.

The relationship between cognitive and neural measures of face processing has been also examined in a study that examined different cognitive measures of face perception and memory and various face-related event-related potential (ERP) components (Herzmann et al., 2010). This study revealed moderate correlations between a cognitive measure of face processing (i.e., a combined performance score on various perception and memory tasks) and the latency of the N170, an ERP component that is much stronger to faces than other stimuli (Figure 2B), but no correlation with an earlier component, the P100. Moderate correlations were also found with later ERP components related to face memory or person recognition. Such studies are important in determining which aspects of face processing are reflected by different ERP components and provide converging evidence to the majority of ERP studies that employ the more common group-based analysis approach (e.g., Schweinberger et al., 2004).

## Do EEG and fMRI measures of face processing reflect the same mechanisms?

Face-selective neural responses (i.e., higher group-mean response to faces than non-faces) have been reported in hundreds of fMRI and EEG studies. However, only one study has examined the correlation across individuals between the EEG and fMRI face-selective measures. This study revealed that the magnitude of face-selectivity in both the FFA and the STS-FA were associated with the face-selectivity of the EEG response approximately 170 ms after stimulus onset (N170) (Sadeh et al., 2010; Figure 2E). The face-selectivity of the occipital face area (OFA) was not correlated with the face-selectivity of the N170 but was correlated with ERP face-selectivity at 100–110 ms after stimulus onset, consistent with transcranial magnetic stimulation studies that varied pulse timing (Pitcher et al., 2007, 2012). These studies nicely demonstrate how correlational analysis of EEG and fMRI can reveal temporal dissociations among different brain regions and link different brain areas to the time course of different stages of face processing. Importantly, these correlations extend group-means findings by showing which of these neural face-selective measures, which have been primarily studied separately, are strongly linked and therefore reflect the same underlying neural mechanisms of face processing.

A similar approach has been used to investigate the face inversion effect present in ERP and fMRI measures. The mid-temporal face-selective areas, the FFA and STS-FA show a higher response to upright than inverted faces. In contrast, object general areas (LOC) show a higher response to inverted than upright faces (Yovel and Kanwisher, 2005). The N170 shows increased and slightly delayed amplitude to inverted than upright faces. Two mechanisms have been suggested to account for the increased N170 amplitude to inverted than upright faces. According to the *qualitative hypothesis* increased amplitude for inverted faces reflects the recruitment of additional non-face mechanisms that are not used for the processing of upright faces. Thus, the increased response to inverted faces in the object area may contribute to the increased N170 amplitude to inverted faces. In contrast, the *quantitative hypothesis* suggests the same processes generate the N170 response to upright and inverted faces but that the increased amplitude for inverted faces reflects the greater demands that inverted face processing places on face mechanisms.

To directly test these two hypothesis, the N170 and fMRI face inversion effects were measured in a simultaneous EEG-fMRI study. The N170 face inversion effect was calculated for each subject as the normalized difference between the response to upright and inverted faces (Sadeh et al., 2011). In addition, face-selective and object general areas were localized and the difference in their response to upright and inverted faces was measured. A correlational analysis between the fMR- face inversion effect (i.e., the difference between the response to inverted than upright faces) in the object and face-selective areas and the N170 face inversion effect revealed a very strong correlation with the object areas (*r* = 0.8) but not with the face areas. These findings further support the *qualitative hypothesis*, which suggests that inverted faces engage object mechanisms that are not used for the processing of upright faces (see also, Moscovitch et al., 1997; Pitcher et al., 2011).

These simultaneous fMRI-EEG studies nicely demonstrate how combining the two methods can provide insight into the temporal characteristics of brain areas and the possible neural generators of ERP signals. In particular, the correlations between the face-selective measures indicate an earlier latency for the OFA than for the mid-temporal face areas. The face inversion effect study attributed the increased amplitude of the N170 to inverted faces, to object areas rather than to the nearby face-area, a finding that cannot be obtained from source localization analysis of EEG data alone. These findings therefore do not only further establish the link between the ERP and fMRI face markers but also enhance our understanding of the spatial-temporal architecture of the face system.

## How specific is face recognition ability?

In the sections above, we have explored how individual differences can link and dissociate mechanisms *within* the domain of face processing. Individual differences can also reveal links and dissociations *between* face processing and other cognitive abilities.

**Figure 3:**
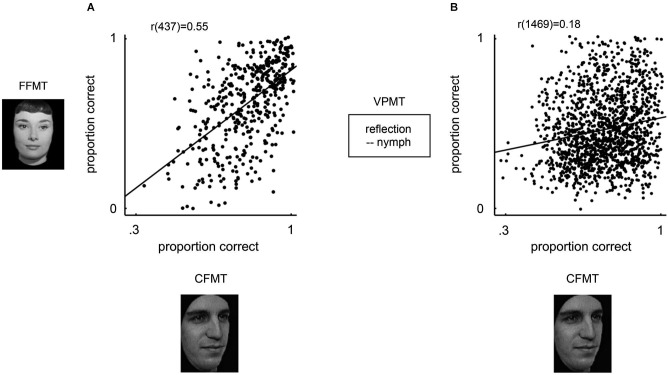
**Specificity of face recognition ability (Wilmer et al., 2012)**. Face recognition performance (x axis, Cambridge Face Memory Test (CFMT)) is plotted against famous face recognition performance (y axis, graph **A**, Famous Faces Memory Test (FFMT)) and verbal recognition performance (y axis, graph **B**, Verbal Paired-associates Memory Test (VPMT)). FFMT and CFMT are very different tests. FFMT measures the ability to name faces stored incidentally in memory over months or years of cultural exposure. CFMT, on the other hand, measures the ability to identify faces stored intentionally in memory shortly before being tested. CFMT and FFMT nevertheless show a high correlation, demonstrating that CFMT captures a general face recognition capacity. CFMT dissociates strongly, however, from VPMT, which measures the ability to identify word-pairs stored intentionally in memory shortly before being tested. This dissociation demonstrates that CFMT captures a specific recognition capacity.

An active line of research has recently revealed that face recognition is an uncommonly specific ability. In the psychometric literature, the term *specific* is typically applied to an ability that shows some degree of independence from general intelligence (Wai et al., 2009). By this definition, face recognition is exceptionally specific. To date, its mean reported correlation with measures of general intelligence, weighted for sample size and corrected for range restriction in the IQ measures, is 0.01 (Davis et al., 2011; Peterson and Miller, 2012; Palermo et al., 2013). Moreover, face recognition dissociates strongly even from other types of recognition memory. For example, in diverse samples totaling over 4000 participants, well-validated tests of verbal and non-face visual recognition ability, respectively, explained only about 3% and 7% of the variance in face recognition ability (Wechsler, 1997; Wilmer et al., 2010, 2012; Figure 3). These findings are consistent with several reports that show that neuropsychological cases sometimes show selective impairment or sparing of face recognition (Moscovitch et al., 1997; Duchaine et al., 2006; Rezlescu et al., 2012; Busigny et al., 2014).

Ironically, the history of psychometric ability testing has seen face recognition ability dropped at least twice from prominent test development efforts when its pervasive dissociations from other social and memory abilities were mistaken for lack of valid measurement (Thorndike, 1936; Kihlstrom and Cantor, 2000; Holdnack and Delis, 2004; Wilmer et al., 2012). Only in recent years has it been discovered that face recognition’s dissociations from other abilities reflect a valid, unique dimension of human ability (Wilhelm et al., 2010; Wilmer et al., 2010, 2012; Hildebrandt et al., 2011; McGugin et al., 2012; Figure 3). Guided by the example of face recognition, we suggest that an individual differences approach be used to further define the cognitive and neural boundaries of face processing, as well as to search for other unique social and cognitive ability dimensions.

## How is face recognition ability shaped by genes and environment?

**Figure 4:**
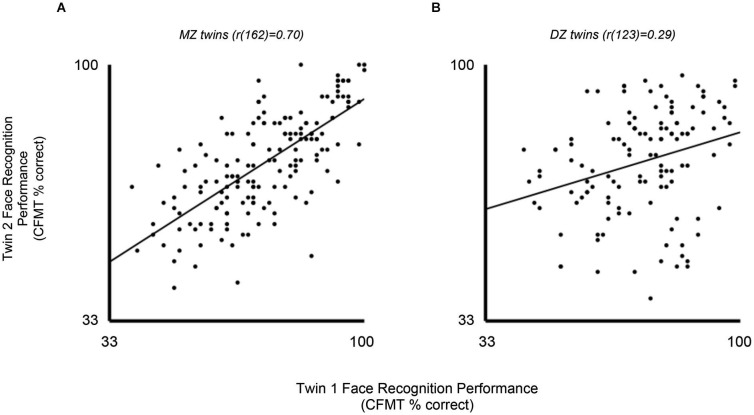
**Heritability of face recognition ability (Wilmer et al., 2010)**. In this study of twins, the second-born twin’s CFMT score (y axis) is plotted against the first-born twin’s score (x axis) for monozygotic (MZ, *n* = 164) twins **(A)** and dizygotic (DZ, *n* = 125) twins **(B)**. MZ correlation is rMZ(162) = 0.70, and DZ correlation is rDZ(123) = 0.29. The high rMZ indicates high family resemblance for CFMT performance, whereas the rDZ of less than half the rMZ indicates that most or all of this family resemblance can be attributed to family genes rather than family environments.

Individual differences in face processing abilities provide a powerful vehicle for exploring the contributions of genes and environments to face processing via twin and family studies. A recent twin study showed that face recognition in adults is highly heritable (Wilmer et al., 2010). Correlations between identical twins on the CFMT (0.70) were twice those of fraternal twins (0.29), evidence that the strong family resemblance for face recognition ability resulted from genetic factors rather than common environmental factors (Figure 4).

The combination of face recognition’s uncommon specificity and high heritability runs counter to a classic finding in behavioral genetics that more specific abilities are less heritable (Plomin et al., 2013; see section above on face recognition’s specificity). That classic finding inspired a prominent theory that the majority of genetic variance in any cognitive ability is attributable to general intelligence (Kovas and Plomin, 2006). Face recognition presents a clear exception to that theory. Further, face recognition’s heritability suggests that it could be used as a model system to study cognitive and neural resilience to environmental variation. Despite its strong dissociation from general intelligence, face recognition may be similar to general intelligence in showing increasing heritability with age (Wilmer et al., 2010; Zhu et al., 2010). If so, then increasing heritability may be a more generalized principle of development than previously recognized.

Future work is needed to determine whether adult face recognition can be parsed into heritable subcomponents. One twin study found a non-zero genetic contribution to the composite face effect, but not to the part-whole effect, suggesting a relatively constrained role of holistic face processing mechanisms in face recognition’s heritability (Zhu et al., 2010). Future work could also explore the specific genetic and environmental mechanisms by which a broad natural tendency for relatively good or poor face recognition ability is expressed. A richer understanding of such mechanisms might inspire novel interventions to enhance, or accommodations to support, the important social task of recognizing others in our everyday lives. Moreover, a more detailed understanding of the reasons for face recognition’s high degree of resilience to environmental variation might fuel efforts to maximize neural and cognitive resilience in other domains as well.

## Conclusion

This review demonstrates how assessment of associations and dissociations among measures of face processing by an individual differences approach can provide answers to basic questions about face processing mechanisms. The questions tackled by the examples in our review address the nature of various face processing mechanisms, their relationship to each other, and their relationship to broader aspects of cognition. Many questions still await such investigations. These questions include: what associations and dissociations exist between additional face processing mechanisms, including those used to glean age, gender, and attractiveness? What are the detailed neural and genetic mechanisms of each aspect of face processing? What plasticity exists, at what ages, and what are the practical correlates good or bad at face processing? How do aspects of face processing beyond face recognition relate to a broader array of human capacities?

Most existing work on individual differences in face processing has aimed to isolate broad patterns of association and dissociation among abilities, or between abilities and their underlying mechanisms. Much work remains to be done at this relatively coarse level of analysis. At the same time, there is a need to begin digging deeper, making increasingly fine-grained theoretical distinctions about the specific neural, cognitive, genetic, and environmental mechanisms that shape and constitute such broad associations and dissociations. Fine-grained work of this sort is both promising and challenging; it requires (a) a greater number of high-quality tests; (b) more highly multivariate statistical models; and (c) a larger number of participant-hours. Each such requirement comes with its own costs and complications, however, all can be overcome for fine-grained questions of sufficient theoretical or practical import.

As this review indicates, correlational analyses not only expand our methodological and statistical armory but also force consideration of the theoretical meaning of our measures in a way that a group-mean approach may not. The individual differences approach can therefore provide valuable information that complements and extends the inferences supported by the commonly used group-mean approach. We anticipate this approach will be as fruitful in other domains of cognitive science as it has been, and will likely continue to be, in the study of face processing.

## Conflict of interest statement

The authors declare that the research was conducted in the absence of any commercial or financial relationships that could be construed as a potential conflict of interest.

## Footnotes

^1^The composite face effect has been measured in these studies with two different paradigms, the *congruency/interference paradigm* or the *standard paradigm*. A comprehensive discussion about the two types of paradigms and the extent to which they provide a valid measure of holistic processing are discussed in Richler and Gautheir (2013) and Rossion (2013). Briefly, the *standard paradigm* provides a measure of holistic processing by assessing the interference of irrelevant different face halves on the processing of the other face halves. The *congruency/interference design* provides a general stroop-like measure in which congruent and incongruent trials are compared.

^2^Regression scores are computed by regressing, rather than subtracting, the part-based condition from the whole face condition. In the part-whole task, regression scores are computed by regressing the part condition from the whole condition. In the composite face task, regression scores are computed by regressing the misaligned task from the aligned task. A more detailed discussion about the subtraction and regression approaches is found in Degutis et al. (2013).
